# Identification and expression analysis of antigenic sites of hepatitis C virus genotype 3a NS3 and NS5A genes of local isolate

**DOI:** 10.1186/s43066-021-00086-8

**Published:** 2021-03-08

**Authors:** Sabeen Sabri, Muhammad Idrees Khan, Shazia Rafique, Amjad Ali, Muhammad Saleem Khan

**Affiliations:** 1 Department of Microbiology and Molecular Genetics, Faculty of Life Sciences, University of Okara, Okara, 56130 Punjab Pakistan; 2 Centre of Excellence and Molecular Biology, University of the Punjab, Lahore, Pakistan; 3 Department of Biotechnology and Genetic Engineering, Hazara University, Hazara, Pakistan; 4 Department of Zoology, Faculty of Life Sciences, University of Okara, Okara, Punjab 56130 Pakistan

**Keywords:** Analysis, Hepatitis C, Genotype, 3a NS3, NS5A, Genes

## Abstract

**Background:**

Hepatitis C virus, a silent killer, has infected 71 million people globally. The recombinant viral antigenic proteins might be used in the early diagnosis of HCV infection. The NS3 and NS5A genes of HCV function in HCV replication and influence host cellular factors that are involved in HCV pathogenesis. The current study was designed to select NS3 and NS5A antigenic sites, amplified, cloned, and expressed in order to find out better assays for diagnosis or drug and vaccine development. The antigenic sites within NS3 and NS5A genes were selected and confirmed through sequencing and were cloned. The antigenic recombinant proteins were expressed in bacterial strain E. coli BL21ply*, and the expression was confirmed by western blotting by using gene-specific and vector-specific antibodies.

**Results:**

Specific antigenic regions within the NS3 and NS5A genes of the HCV 3A genotype were amplified. PCR results showed 328 bp and 747 bp antigenic regions, respectively. The regions were confirmed by DNA sequencing and cloned into a bacterial expression vector. Expression analysis showed 12 kDa and 28 kDa of NS3 and NS5A antigenic recombinant proteins, respectively. Taken together, these studies will help to analyze the genetic variability within the local HCV isolates as these antigenic recombinant proteins were quite important in the screening of HCV-infected patients.

**Conclusions:**

This study might help to enhance the progress in the treatment of HCV infection through the modeling of HCV non-structural genes (NS3 and NS5A) from local isolate, and it might also present the viral genes as potential therapeutic targets.

## Background

The basic structural research on designing new therapeutic drugs against the silent killer (HCV) enhances the management of hepatitis C virus infection [1–4]. The prompt and efficient diagnosis of HCV is quite useful for the cure of the disease manifestation [5–8]. Concerning clinical data, it is perceived that antibody response against certain HCV proteins was quite variable. The immunogenicity of hepatitis C proteins varies as some of the proteins are highly immunogenic while others are not [9–11]. The serological diagnosis of HCV is mainly done by using the specific antibody through the enzyme immunoassays (EIAs) [12, 13].

Along with the routine diagnostic tests, some new methods are required for the selection of sequence variants of antigens that can develop antibodies against the diverse genotypes of HCV. Recombinant HCV proteins are most suitable for the study of the antigenic heterogenecity of the proteins. The available data showed that the determined antigenic regions of HCV possess sequence heterogeneity which influences the antigenic properties of these antigenic regions [14]. Keeping this in view, the antigenic sites of NS3 and NS5A genes of HCV genotype 3a from the local isolate were determined, amplified, and cloned into the bacterial expression vector. The antigenic recombinant proteins were expressed in the bacterial strain E. coli BL21ply*, and the expression was confirmed through western blotting by using gene-specific and vector-specific antibodies.

## Methods

### Synthesis of cDNA and amplification of NS3 and NS5A genes of HCV3a

After the identification of chronically infected HCV genotype 3a patient from the Division of Molecular Virology and Molecular Diagnostics, the cDNA was synthesized of the extracted RNA as described [15]. The synthesized cDNA was used as a template for the full-length amplification of the NS3 and NS5A genes of HCV 3a genotype of the local isolate using gene-specific primers [15]. The amplified products of NS3 and NS5A were submitted to GeneBank, and accession numbers were obtained.

### Identification of antigenic sites within the NS3 and NS5A genes

From the confirmed sequences of NS3 and NS5A genes of HCV 3a genotype, the antigenic sites were selected by using the software Antigenicity Plot ((http://www.bioinformatics.org/JaMBW/3/1/7/). The selected antigenic regions of NS3 and NS5A genes were designated as NS3.1 (nt ~ 328 bp) and NS5A.1 (nt ~ 747 bp) and were used as a template.

### Amplification of the NS3.1 and NS5A.1 antigenic sites

To amplify the antigenic sites NS3.1- and NS5A.1-specific primers containing the restriction enzyme, sites were designed. For NS3.1 primers, *HindIII* and *XhoI* restriction enzyme sites were added to the primers while NS5A.1 primer contained *EcoR1* and *HindIII* restriction enzyme sites.

### Construction of expression vector containing NS3.1 and NS5A.1 antigenic sites and bacterial transformation

To construct an expression vector containing the NS3.1 and NS5A.1 antigenic regions, both the amplified sites and vector were treated with restriction enzymes. For the construction of the NS3.1 expression vector, the gene and vector were treated with *HindIII* and *XhoI* restriction enzymes. In order to construct the NS5A.1 vector, the amplified antigenic site and vector were treated with *EcoR1* and *HindIII* enzymes. The digested genes were ligated into the digested plasmid to construct the vector expressing the NS3.1 and NS5A.1 antigenic regions of HCV 3a. After the confirmation of correct ligation by sequencing 2 μl of plasmids harboring the antigenic regions (NS3.1 and NS5A.1) of NS3 and NS5A, genes of HCV 3a genotype of the local isolate were transformed into competent BL21 DE3 pLysS bacterial cells by heat shocking for 90 s at 42 °C. LB medium of 500 ml free of antibiotics was added and incubated for 1 h at 37 °C. Selection of transformants of NS3.1 and NS5A.1 was done on LB-agar plates containing kanamycin (25 mg/ml) and chloramphenicol (34 mg/ml) and incubated overnight at 37 °C.

### Expression studies of NS3.1 and NS5A.1 antigenic sites

The expression of the antigenic sites of the NS3 and NS5A genes of the HCV 3a genotype was determined by western blotting. Individual pET28 clones NS3.1-28 and NS5A.1-28 was transformed into the bacterial strain by using the heat shock method as mentioned previously; 2 μl of plasmid was transformed into the BL21 (DE3) pLysS, and selection was done on L-agar plates supplemented with chloramphenicol (34 mg/ml) and kanamycin (25 mg/ml). Isolated colonies were selected and inoculated into L-broth supplemented with chloramphenicol and kanamycin and incubated at 37 °C overnight on a shaker.

### Western blot analysis

The confirmed clones (colony PCR) were then used for expression analysis. Individual colonies were given induction of 0.5 M IPTG for 4 h. For protein, isolation cells were harvested, and for 1 g of pellet, 10 ml of lysis buffer was added. Thaw the pellet at room temperature. Prepare a protein sample by mixing 65 μl of the sample with 35 μl of 6X protein loading dye. This is the sample of the total extract. Now, add 0.004 g of lysozyme to the remaining extract and mix well. Sonicate (Mixsonic, USA) the lysed cells for 15 s × 4 pulses. Transfer the pellet into a separate tube and wash the pellet with three times with lysis buffer. Now resuspend the pellet 400 μl of 1X PBS. Prepare the protein sample by mixing 65 μl of pellet and 35 μl of 6Xprotein loading dye. Heat shock for 7 minutes in boiling water bath and snap cool on ice for 5 min. Run-on 15% SDS-PAGE gel at 60 V for 90 min. Western blot was performed following [15] using the gene-specific and vector-specific His tag antibodies.

## Results

### Selected antigenic site of NS3 and NS5A genes

**Fig. 1:**
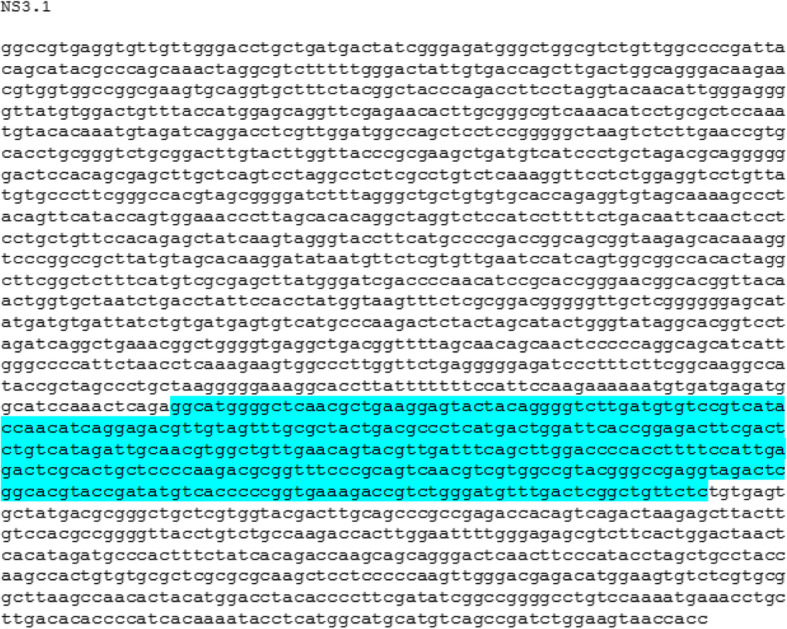
Full gene sequence of NS3 gene indicating (highlighted) specific antigenic site NS3.1

**Fig. 2:**
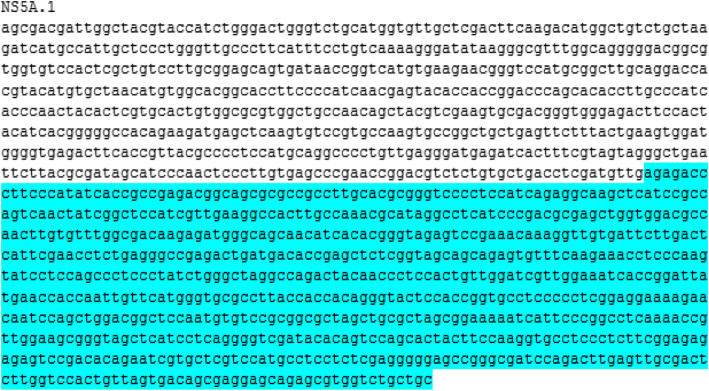
Full gene sequence of NS5A gene indicating (highlighted) specific antigenic site NS5A.1

To determine the antigenic regions of the NS3 and NS5A genes, antigenicity plot was used. The full sequence of NS3 and NS5A genes was analyzed for the analysis of the antigenic sites. Figure 1 illustrates the antigenic sites NS3.1 determined in the NS3 gene of the virus. In Fig. 2, the determined antigenic site NS5A.1 of NS5A gene of the virus is shown.

### PCR amplification of antigenic site

**Fig. 3:**
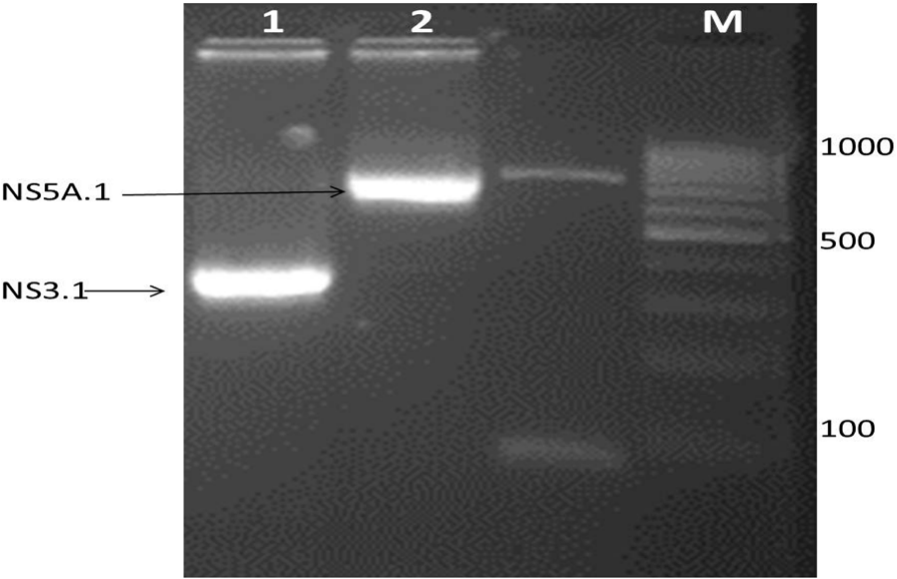
PCR amplification of antigenic sites (NS3.1 (328 bp) and NS5A.1 (747 bp)) using antigenic restriction site-specific primers. Lane 1: NS3.1-amplified antigenic site (328); lane 2: NS5A.1-amplified antigenic site (747); M: 100 bp marker

**Fig. 4:**
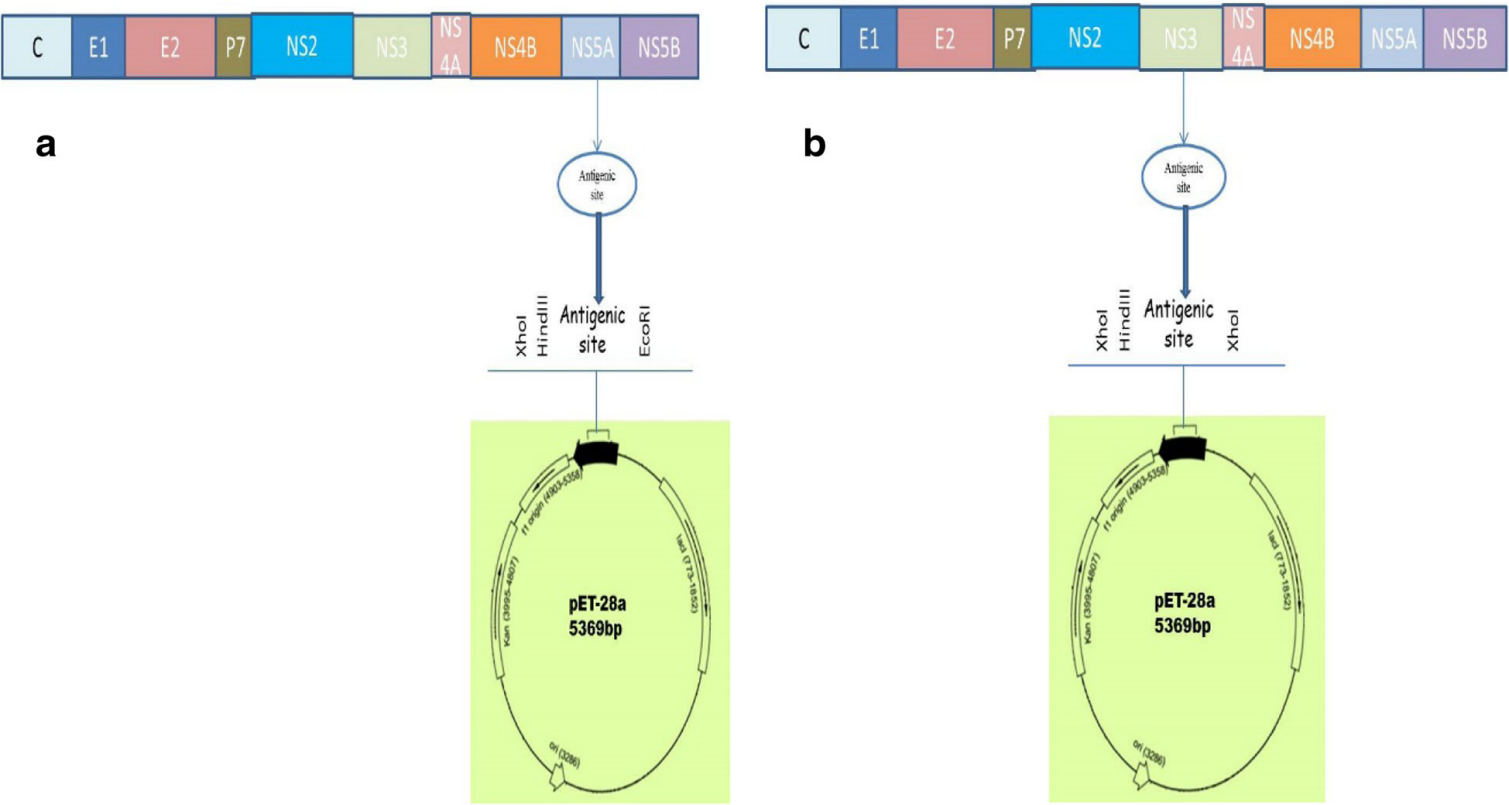
pet28a constructs of NS3.1 (**a**) and NS5A.1 (**b**) encoding antigenic sites

To clone the antigenic site regions into the pET-28a vector the determined antigenic regions NS3.1 and NS5A.1 were PCR amplified by utilizing the antigenic site-specific restriction primers. Designed primers for NS3.1- and NS5A.1-specific antigenic sites having a start codon ATG and specific restriction sites were synthesized. Figure 4 revealed the amplified regions of the specific antigenic site NS3.1 of the NS3 gene. The expected band size of NS3.1 was 328 bp which was observed on the gel. In Fig. 3, 747 bp gene band of the NS5A.1 antigenic region was observed. This indicates that the determined antigenic sites were successfully amplified. The PCR amplified products were observed onto 2% agarose gel which is stained with ethidium bromide. Both the amplified products were confirmed by restriction digestion and sequencing.

### Cloning of the amplified antigenic sites into the pET-28a vector

To check the expression of the antigenic recombinant clones of NS3.1 and NS5A.1, these amplified antigenic sites were cloned into the pET28a vector. The results were as shown in Fig. 4.

### Restriction and digestion analysis and sequencing confirmation

**Fig. 5:**
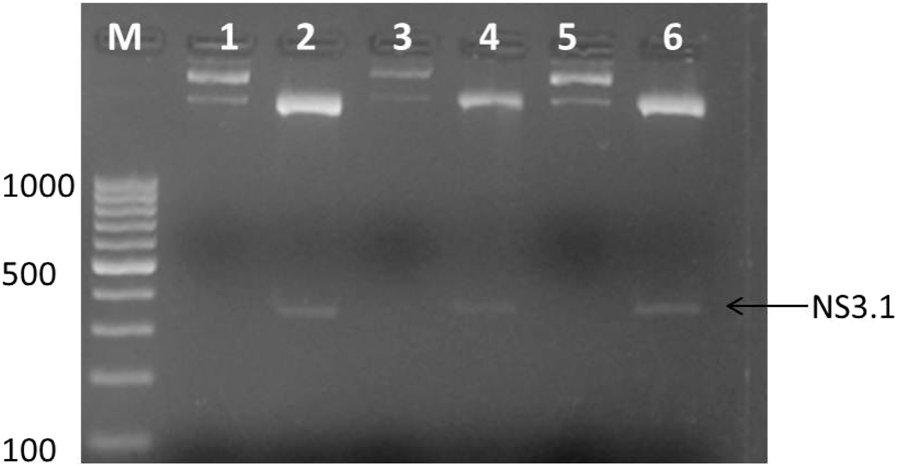
Restriction and digestion analysis of pet28a vector expressing NS3.1 antigenic sites. Lanes 1, 3, and 5: uncut vectors; lanes 2, 4, and 6: positively NS3.1-digested plasmids; M: 100 bp marker

**Fig. 6:**
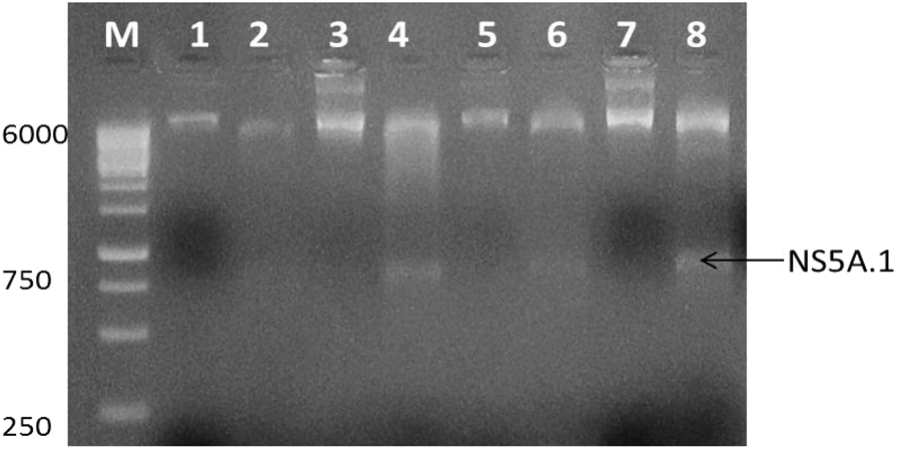
Restriction and digestion analysis of pet vector expressing NS5A.1 antigenic site. Lanes 1, 3, 5, and 7: uncut plasmids; lanes 4, 6, and 8: positively digested plasmids; M: 1 kb marker

The antigenic recombinant clones NS3.1-28 and NS5A.1-28 that showed positive results for plasmid PCR were further confirmed by restriction and digestion analysis. The double digestion of NS3.1-28 clones was done with *HindIII* and *XhoI* restriction enzymes. The results are shown in Fig. 5 confirm the presence of the desired gene of interest with the vector backbone. The restriction and digestion analysis showed an exact product of 328 bp of the NS3.1 region. Lanes 2, 3, 4, and 6 showed positively digested NS3.1-28 antigenic recombinant clones. Figure 6 showed the restriction and digestion analysis of the NS5A.1-28 antigenic recombinant clone. NS5A.1-28 was digested with *EcoRI* and *HindIII* enzyme which gives the required product of 747 bp. The figure shows that lanes 4, 6, and 8 showed positively digested NS5A.1-28 antigenic recombinant vector. These results indicate that the required NS3.1 and NS5A.1 antigenic regions were successfully cloned into the pET28a vector. The dye termination sequencing method was used on an automated sequencer. The sequencing of the positively digested NS3.1-28 and NS5A.1-28 vectors was done by using a T7 vector-specific primer. The results indicate that the amplified antigenic sites NS3.1 and NS5A.1 were successfully ligated in the correct orientation in the pET28a vector. The sequences were BLAST and compared with the known sequences of the 3a genotype. The comparison results indicated that the cloned antigenic sites were of NS3 and NS5A genes of hepatitis C virus genotype 3a of Pakistani isolate.

### Production of antigenic recombinant proteins in bacterial cells

**Fig. 7:**
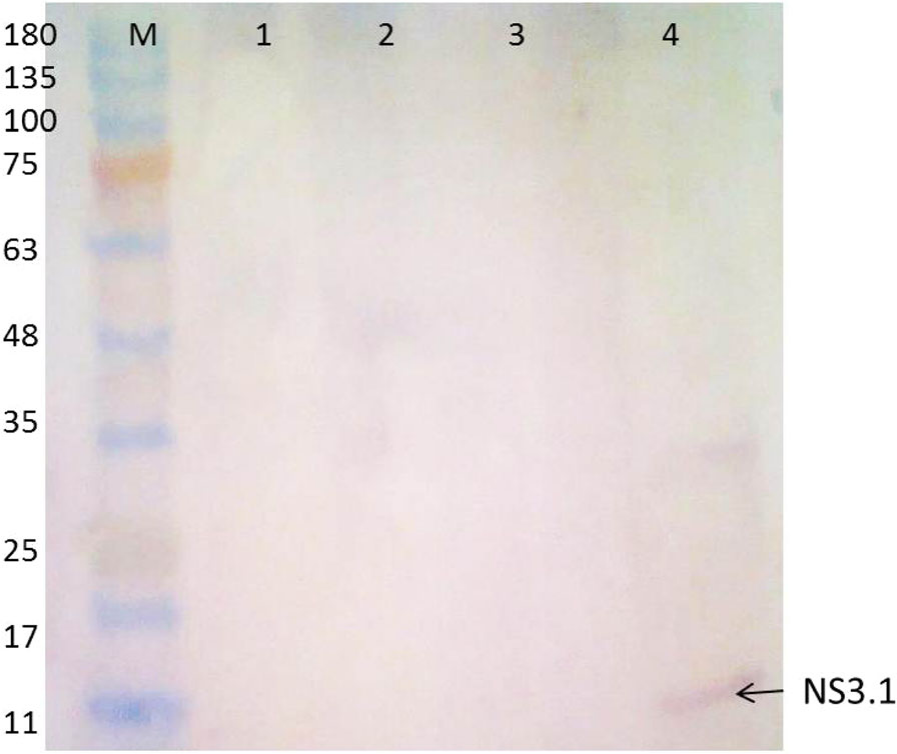
NS3.1 antigenic recombinant protein expressed in bacterial cells (gene-specific antibodies). Lanes 1–4: NS3.1 protein; lane 4: positive NS3.1; M: prestained protein marker.

**Fig. 8:**
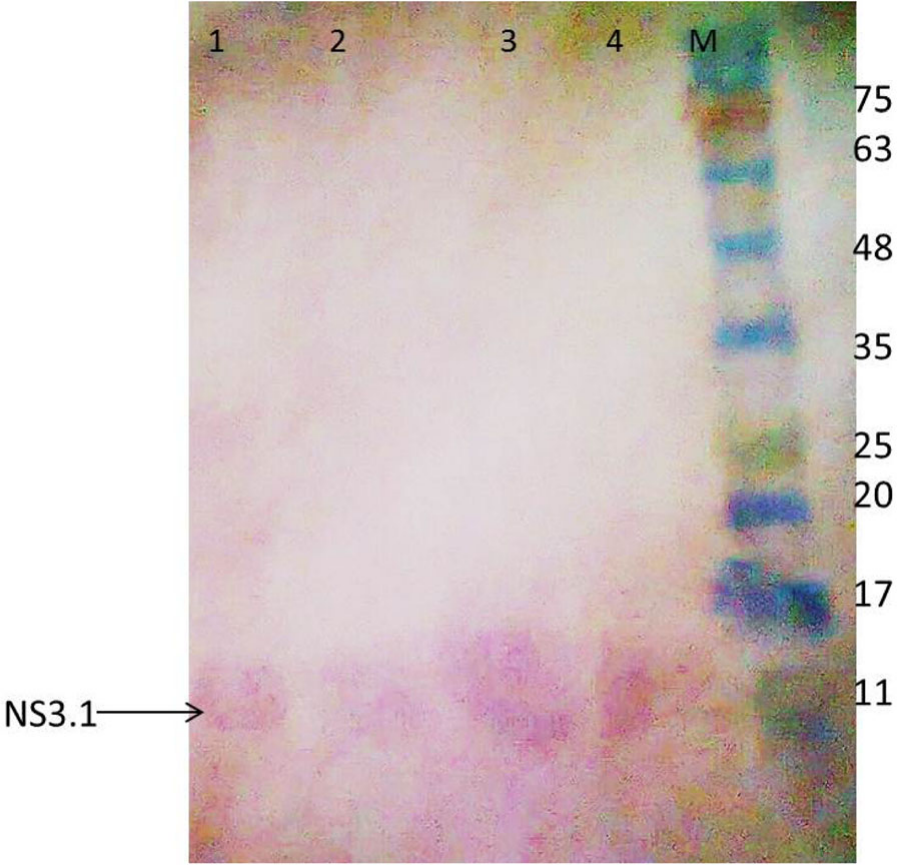
NS3.1 antigenic site protein expressed in bacterial cells (vector-specific antibodies). Lanes 1–4: NS3.1 protein; M: prestained protein marker

The expression of the NS3.1-28 and NS5A.1-28 antigenic replicons was checked in *E. coli* BL21 ply* bacterial strain. The confirmed antigenic recombinant clones were used for expression studies. To characterize the antigenic recombinant protein, the confirmed plasmids NS3.1-28 and NS5A.1-28 were transformed into the *E. coli* BL21 ply* strain. Isolated colonies were selected to check the expression of the antigenic recombinant vectors. The individual colonies were grown overnight in L-broth supplemented with chloramphenicol (34 mg/ml) and kanamycin (25 mg/ml). An induction of 0.5 M IPTG was given for fours. The un-induced culture was used as a control. Protein was extracted after induction, and samples were identified through western blot by using gene-specific as well as vector-specific antibodies. Results shown in Figs. 7 and 8 demonstrated that the expected NS3.1 protein of 12 kDa was observed with both gene and vector-specific antibodies.

**Fig. 9:**
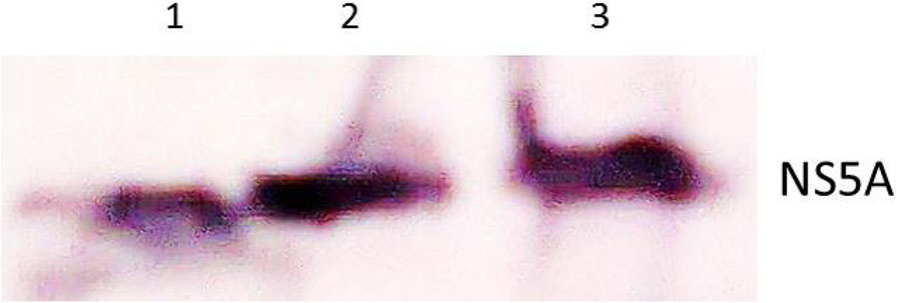
NS5A.1 antigenic site protein expressed in bacterial cells (gene specific). Lanes 1–3: NS5A.1 antigenic protein

**Fig. 10:**
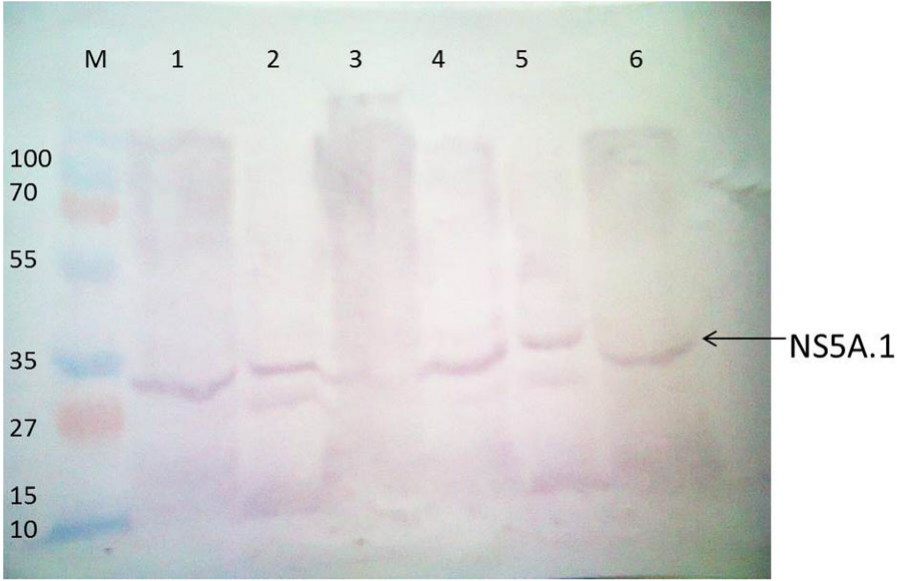
NS5A.1 antigenic site protein expressed in bacterial cells (vector specific). Lanes 1, 2, 4, and 6: NS5A.1 antigenic protein; M: prestained protein marker

The results of NS5A.1 expression were analyzed which showed approximate bands 28 kDa with gene-specific and vector-specific antibodies (Figs. 9 and 10). These results indicated that the amplified antigenic sites of NS3 and NS5A HCV 3a genotype genes were successfully cloned into a bacterial expression vector and expressed well in the bacterial system. These antigenic recombinant proteins were quite important in the screening of HCV-affected patients

## Discussion

The antigenic site is the antibody binding region. It is the hydrophobic part on the surface of the protein molecule which serves as a receptor for the antibody molecule. The determination of antigenic sites is important because a particular antigen triggers the immune response against a particular pathogen. The study of the antigenic sites of the non-structural genes NS3 and NS5A of hepatitis C virus 3a genotype of local isolate might help in designing specific synthetic peptides and development of prophylactic vaccines or enhancing therapeutic drugs or vaccines against the hepatitis C virus. It may also be used to characterize the defensive response against the hepatitis C virus. The determined antigenic regions might prove useful for the development of new screening methods for HCV as well.

The regimens for HCV management mainly involved an early diagnosis of HCV infection. The quantification of HCV antigens (Ag) has been used for viral detection which also serves as a substitute for viral load estimation [16]. The determination of an appropriate antigenic site or epitope region is a prerequisite for the development of diagnostic approaches, medicines, and therapeutic drugs [17]. To improve the routinely used diagnostic procedures for HCV infection and to thwart the spread of this prevailing infection in the local population, this study was designed to determine the immunogenic or antigenic regions within the HCV non-structural genes (NS3 and NS5A). The determined antigenic regions are of high significance as these are implemented in ELSA-based diagnostics tests for HCV infection which gives more reliable and less false-positive results. The generated recombinant proteins might be used to develop screening assays for HCV infection and to develop certain vaccines. The available data also showed that the immunogenic region within the HCV genes is valuable to establish new screening techniques and the development of new therapeutic drugs [14, 16, 17]. Moreover, these results are also beneficial to analyze the genetic variability within the HCV non-structural genes (NS3 and NS5A) of Pakistani isolate which might be significant in HCV treatment through modeling of the HCV recombinant antigenic proteins.

## Conclusion

This study could help to enhance the progress in the treatment of HCV infection accompanied by the modeling of HCV non-structural genes (NS3 and NS5A) from a local isolate. It may also help characterize the viral genes as potential therapeutic targets for antiviral drugs or prophylactic vaccines.

## Data Availability

Not applicable

## Abbreviations

ATG: Adenine thymine guanine (methionine, a start codon)
BL21DE3: An *E. coli* strain
cDNA: Complementary deoxyribonucleic acid
DNA: Deoxyribonucleic acid
EIAs: Enzyme immunoassays
ELSA: Ethical, legal and social aspects
HCV: Hepatitis C virus
IPTG: Isopropyl β-D-1-thiogalactopyranoside
LB medium: Luria Bertani medium
MIK: Methyl isobutyl ketone
NS3 gene: Hepatitis C virus non-structural protein 3 (HCV NS3)
NS5A: Nonstructural protein 5A (NS5A)
PCR: Polymerase chain reaction
RNA: Ribonucleic acid
ss: Single strand/single-stranded
SDS-PAGE: Sodium dodecyl sulfate polyacrylamide gel electrophoresis
